# Stammering—Its Relation to Poverty and Its Treatment

**Published:** 1884-06

**Authors:** 


### Selections.

### Stammering—Its Relation to Poverty and Its Treatment.

Dr. Barkhan, of Brunswick, Germany, obtained from the teachers in the public schools a list of all the stammerers, and then he examined them singly; there were 86 cases, belonging unexceptionally to the lower classes, laborers and mechanics. All these attended the lower grade schools. There were 72 boys and 14 girls. Most of them were poorly fed and clad, only four presenting a better physical appearance. In 24 cases there was an abnormal relation between the form of the head and the dimensions of the chest. The smallest head was 48 ctm., the largest 57 ctm. The smallest chest 51 ctm., the largest 75 ctm. in circumference. Normally, the circumference of the chest exceeds that of the head 8—11 ctm., rarely 6—8 and 11—13. In the formation of the palate there were various anomalies ; the great depth, and its conjugation with the form of the maxillary, and position of the teeth. He does not regard these as the cause of stammering, but only as an arrest of development, which is often found with idiots and deaf-mutes, which is only a proof of their close relations. There was no evidence of heredity in any of these cases. He proposes a plan for their education which will require six months, three hours weekly.

The 37 worst cases—boys—will require three teachers, who must treat them with the utmost kindness and consideration. A physician should be in constant attendance with the teachers.

*Instruction for Stutterers.*—The first and second months comprise exercises in breathing. 1. 15 minutes. Inspiration and retention of breath, at the same time placing the right hand under the left false ribs, for three seconds, gradually rising to 20 seconds ; repeat this three times, and then rest one minute. Expiration three seconds, increase to ten seconds ; repeat three times, then rest one minute. Then keep time in inspiration and expiration ; pause more or less. This must be done with absolute regularity for several minutes. Beat time like in music, accelerate or retard the breathing, pause, repeat, and proceed. 2. 15 minutes, singing. 3. 15 minutes marching and calesthenics, especially of the arms.

The third and fourth months comprise exercises of the voice. 1. 15 minutes. Repeat breathing exercises—pause 15 minutes. 2. 15 minutes. Deep inspiration, and at expiration give utterance to the vowels, a, e, i, o, u, first deep, then middle, then high, first 5 seconds, then rising to 20 seconds. Later, sing scales, hold the notes long, gradually expand, and then diminish. 3. 15 minutes marching, calisthenics of the arms.

Fifth and sixth months. Reading and speaking exercises. 1. 15 minutes breathing and vocal exercises. 2. 15 minutes exercises in joining vowels to consonants, and then after the consonants, always to be preceded by a deep inspiration. Then beating time to slow speaking. Deep inspiration before every sentence. 3. 15 minutes, beating time to slow reading ; inspiration as before.

*Instruction for Stammlers or Stammerers.*—The teacher must accurately describe the position and motions of the organs of speech which are required for the proper articulation of the faultily pronounced letter or syllable, and practice it until it is corrected; repeat it singly or in combination with other letters or words; also the shape of the mouth at the vowels, the position of the lips at the consonants, the position of the lower lip and upper teeth at s, c, z, etc.

The tongue must never protrude between the teeth in speaking. —*Arch. d. Psychol.*—*St. Louis Med. and Surg. Jour.*

